# The Influence of Combined Sterilization Factors on the Structural and Functional Characteristics of Bone Implants

**DOI:** 10.3390/ijms241914426

**Published:** 2023-09-22

**Authors:** Nadezhda Nikolaeva, Vladimir Rozanov, Alexander Chernyaev, Igor Matveychuk, Milena Makarova

**Affiliations:** 1 Radiation Technologies Laboratory, Institute of Physics and Technology, M.K. Ammosov North-Eastern Federal University, 677000 Yakutsk, Russia; larsoon696@mail.ru (N.N.); a.p.chernyaev@yandex.ru (A.C.);; 2 Physical Faculty, Lomonosov Moscow State University, 119991 Moscow, Russia; 3 Scientific and Educational-Methodical Center of Biomedical Technology, The All-Russian Research Institute of Medicinal and Aromatic Plants, 117216 Moscow, Russia; mivilar@mail.ru

**Keywords:** bone tissue, ozone, radiation sterilization, IR spectroscopy, scanning electron microscopy, elemental analysis, microhardness

## Abstract

The results of a comprehensive study of the patterns of structural and functional changes in bone tissue samples after combined (ozone + radiation) sterilization are presented. The study used a different approach to the sterilization process with selective ozone or radiation exposure and an integral, combined one, based on a combined ozone–oxygen treatment of bone samples at the first stage and radiation at the second. The methods of IR spectroscopy, scanning electron microscopy with a prefix for elemental analysis, atomic force microscopy, and mechanical analysis with determination of elastic-plastic properties (Vickers microhardness index) were used in the work. It is shown that the ozone exposure used at the first stage of the combined sterilization process of bone implants does not lead to negative consequences with respect to their properties and characteristics. The results obtained serve as a scientific and methodological basis for the further improvement and optimization of sterilization technologies (including combined). They also offer a comprehensive justification of the parameters of sterilization regimes to ensure the safety of using bone implants during reconstructive operations, minimizing structural and functional changes in bone matter, and creating effective health-saving technologies and the possibility of using them for various biomedical applications.

## 1. Introduction

Currently, the need for plastic materials for reconstructive and reconstructive operations has increased significantly [1]. This trend is explained by the high level of diseases of the musculoskeletal system, industrial and transport injuries, an increase in the number of operations related to bone grafting, etc. An urgent problem that also requires attention and solution is the analysis of the consequences after the coronavirus pandemic. At the same time, bone fragility is noted due to a decrease in calcium absorption and its delivery to the bones [2]. The incidence of aseptic necrosis after COVID-19 and the number of endoprosthesis surgeries have sharply increased.

To solve these problems, the factors determining the effectiveness of the manufacture of bioimplants, their sterilization and storage have been analyzed and established. According to the World Health Organization, about two billion people in the world suffer from diseases of the musculoskeletal system. Over the past decade, the number of transplant procedures has increased significantly. According to the Russian Ministry of Health, about five million operations on the musculoskeletal system are performed annually, and almost half of them use bone implants.

At the same time, the biological materials for the implants used during operations must meet strictly established requirements, and, first of all, ensure a high level of safety, excluding the possibility of introducing any infections into the patient’s body. Currently, in order to meet the increased needs of medical institutions for tissue transplants, specialized tissue banks have been created in many countries that are engaged in harvesting, preservation, sterilization and storage of tissues. In Russia, increased attention is paid to the improvement of bone implant manufacturing technologies and the creation of modern plastic materials, ensuring the safety of tissue bank personnel when working with the material, the doctors in the operating room during bone grafting, the recipient himself during transplantation, etc. These factors are largely determined by the quality of sterilization. In this regard, the creation and application of new approaches and solutions, such as Nanofiber scaffolds based on extracellular matrix for articular cartridge engineering [3], as well as various nanomaterials (see, for example, [4,5]), characterized in some cases by pronounced antibacterial activity [6,7], do not detract from the importance of effective sterilization and even make such developments more relevant and in demand.

The main methods of implant sterilization are steam, chemical (using special solutions), gas (ethylene oxide), radiation (by a stream of gamma quanta or fast electrons), etc. [8]. During steam treatment, heating occurs, which negatively affects temperature-sensitive materials, including bone tissue. Gas sterilization (formaldehyde, ethylene oxide) is associated with the use of toxic and even carcinogenic chemicals.

There are also new developments offering to use various physical and chemical factors and their combinations for sterilization. An example is the use of powerful electromagnetic pulses of the microwave range of nanosecond duration, providing a non-thermal mode of exposure [9]. Another approach is the development of the technology of sterilization with peracetic acid in ethanol (Peracetic acid–ethanol sterilization) [10]. Previously, a combined method was proposed in which radiation treatment with deep programmed cooling (crioradiosterilization with programmed freezing technology) was used [11]. There is a positive experience of sterilization of bone implants using liquid media [12], indicating that in these cases effective sterilization was accompanied by the preservation of the initial morphomechanical characteristics of bone fragments.

Other methods of implant sterilization using microwave, ultraviolet irradiation, low-temperature plasma, cryopreservation of drug saturation, etc., are also being developed [8,13,14,15].

Radiation treatment is considered the most effective method [13]. However, a significant disadvantage of the method is the strong influence of radiation on the characteristics of implants, for example, on the structure of bone fragments, their biophysical and mechanical properties, as well as the presence of a number of dose-dependent effects that can lead to the deterioration of the properties and characteristics of bone material [16]. Among the technologies of sterilization of bone implants, the radiation method of processing bone fragments with a stream of gamma photons has been widely used. The determining parameter for radiation sterilization is the amount of absorbed dose. Its generally accepted value, established by the IAEA for the sterilization of bone implants, is 25 kGy. However, when using such a standard dose, negative side effects occur in the bone material (a decrease in the bioplastic properties of the implant, inactivation of morphogenetic proteins, etc.). With an increase in the absorbed dose, the mechanical characteristics of the bone tissue also change. With an increase in the dose within (25–50) kGy, the strength during the bending test, for example, can decrease by almost a third [16]. Changes in stiffness and elongation of the samples are less pronounced, although for such grafts as the anterior cruciate ligament, this may be of critical importance. High radiation doses can lead to the deterioration of the surface quality of the bone implant, changing its structure, mechanical characteristics and the elemental composition of the surface layer [17].

One of the significant negative factors may be the degradation of collagen, which is considered as one of the reasons for the decrease in the quality of the bone implant [18].

In this regard, it is of particular interest to develop and substantiate the applicability and experience of practical use of combined radiation technologies for the sterilization of bone bioimplants. Recent studies show that the achievement of such a result can be ensured by the use of sterilization techniques based on the combined effect of physical and chemical factors on the sterilized plastic material. With the mutual strengthening of the sterilizing effect of these factors, prerequisites are created for the synergistic effect of their action, and the intensity of the impact of each of the factors can be reduced. This makes it possible to reduce the degree of harmful side effects of each of them separately while enhancing the overall effect.

In one of the first works [19] in which the authors implemented such an approach, a two-stage sterilization process was proposed. At the first stage, the bone implant was placed for 24 h in an aqueous solution containing ethanol, dimexide, and thymol at a certain ratio of components. Then, the transplants in sterile packaging were frozen at −70 °C and exposed to gamma rays. As a result, it was possible to obtain a sufficient level of sterility of the samples at the absorbed dose value of 12 kGy. However, the long duration of the process, as well as the high water content in the bone fragment after the first stage, which can lead to the undesirable side process of hydrolysis under radiation exposure, are significant limitations to the applicability of this technological approach.

In recent years, a new combined sterilization method using ozone exposure and radiation exposure has been proposed [20]. The pronounced bactericidal properties of ozone have been known for a long time and have found application in many areas, starting with water purification. The drinking water ozone treatment plant, which provided a high degree of purification and the absence of residual carcinogenic substances, was first built in Monaco at the beginning of the XX century. The first applications of ozone technologies were precisely as a sterilizing agent for the disinfection of operating rooms. An important feature of this use of ozone is the ability to effectively neutralize a variety of bacteria, viruses, fungi, etc., even with short-term exposure to ozone–air mixtures of low concentrations [21].

The technology proposed by the authors of [20] also assumes a two-stage process: at the first stage, the bone sample is treated with an ozone–oxygen mixture with a concentration of (6–8) mg/L. As a result, due to the high bactericidal ability of ozone (even in such small concentrations), such exposure leads to a weakening of the pathogen population and a significant decrease in their radioresistance. As a consequence, during radiation treatment at the second stage of the sterilization process, it is possible to achieve a high level of sterilization with a decrease in the absorbed dose to 11–12 kGy. The results obtained make it possible to use a new promising technique for processing bone materials with the minimal possible changes in their structure, properties and characteristics, to optimize the parameters of the sterilization process of samples.

At the same time, any new technology requires a comprehensive study of the features and consequences of both selective and combined effects of all active factors. The effects of radiation exposure have been studied within a wide range of changes in the absorbed dose, but the possible consequences of ozone exposure have not been sufficiently investigated.

In this regard, the purpose of this work was to study the effects of selective ozone and combined (ozone + radiation) effects on the properties and characteristics of bone fragments during combined sterilization.

## 2. Results and Discussion

In the course of the studies of the properties and characteristics of the bone implant surface after various types of sterilizing effects, comprehensive information was obtained, in particular, on the morphology and chemical composition of the bone sample surface [22].

**Figure 1:**
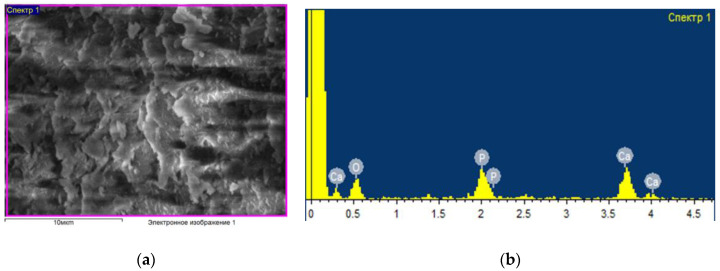
Surface characteristics without ozone treatment. SEM image of bone specimen surface (**a**). Elemental composition on the surface of bone sample (**b**). electronic image 1, Scale bar: 10 µm.

**Figure 2:**
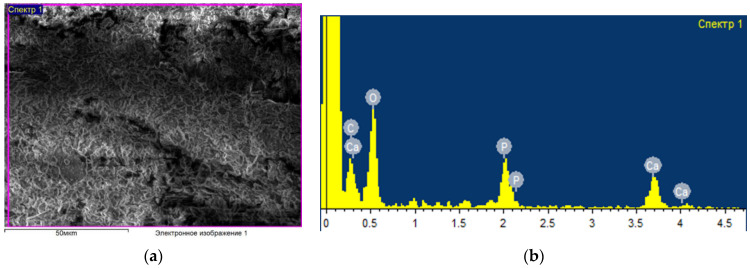
Surface characteristics after ozone treatment (8 mg/L, 15 min). SEM image of bone specimen surface (**a**). Elemental composition on the surface of bone sample (**b**). electronic image 1, Scale bar: 50 µm.

Figure 1 shows an example of the data obtained (micrography of the surface and elemental composition) for a control sample that was not subjected to any processing and was packaged in an unsterilized thermal film. As a result of elemental analysis, the following main chemical elements were identified in the bone composition: oxygen, carbon, phosphorus, sodium and calcium. Other experimental samples (see Figure 2) were treated with an ozone–air mixture with a concentration of (6–8) mg/L for (2–35) min, followed by sealed packaging in thermal film. In this case, there were changes in the percentage of the content of elements; in particular, the concentration of oxygen increases, which indicates a strong interaction with oxygen in the bone composition. At the same time, ozone sterilization does not affect the condition of the surface of the bone sample; no significant structural changes in the surface layer and the percentage of elements other than oxygen have been found.

**Figure 3:**
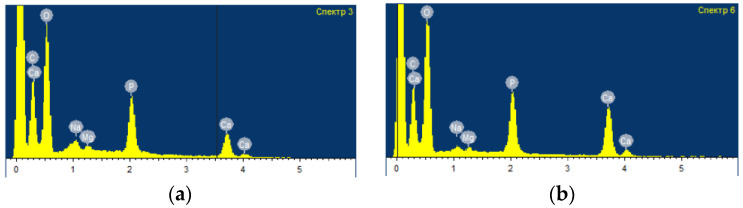
Elemental composition on the surface of bone samples after ozone treatment at a concentration of (8.0–8.5) mg/l. for a different time period: (**a**) 5 min. Full scale 1270 imp.; (**b**) 10 min. Full scale 1489 imp. Full scale 1270 imp.; keV.

The elemental composition of the bovine bone tissue samples treated with ozone for different periods of time was studied. There were no significant differences in the elemental composition of the surface of the samples depending on the time of ozone treatment (Figure 3).

**Figure 4:**
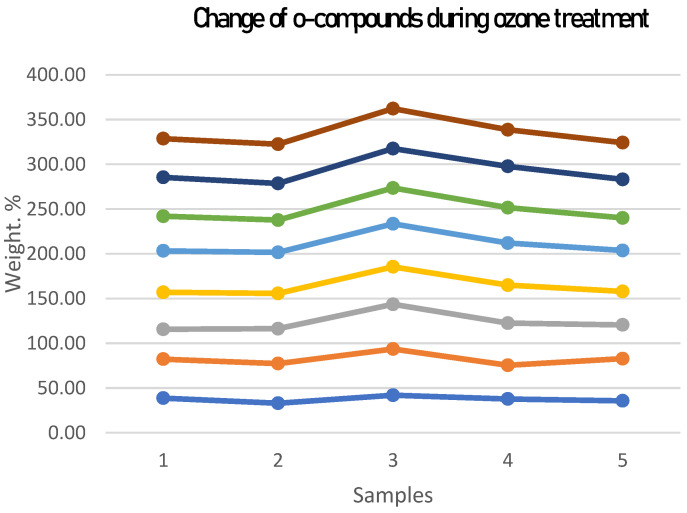
Oxygen concentration in the surface layer of the bone sample at different durations of ozone treatment.

At the same time, experiments have shown that the oxygen content in the surface layer of all the samples studied (see Figure 4) monotonically increases with increasing exposure time to ozone. At the same time, there are no significant morphological changes in the surface. The analysis also revealed the presence of some other elements: carbon, oxygen, phosphorus, calcium, copper, zinc, sodium, magnesium, and silicon. The study of the dynamics of their relative concentration under different processing conditions and the identification of relevant patterns should be the subject of further research.

The use of IR spectroscopy allowed us to obtain new results regarding changes in bone collagen under the sterilizing combined effect. The special attention paid to collagen in this work is due to the fact that it is exposed to destructive effects during radiation sterilization when the absorbed dose exceeds 15 kGy. At the same time, it is collagen that is an important structure and the most common protein in the body of animals and humans, and has great biomedical significance. Collagen is part of the skin, bones and tendons. Collagen-based materials are widely used in medicine, tissue engineering, manufacturing, etc. One of the main disadvantages of collagen biomaterials is their instability. Collagen is destroyed in the body by the action of proteolytic enzymes, such as collagenase. To slow down the biodegradation of collagen-based biomaterials and give them additional strength, various methods of chemical crosslinking of collagen are used, the most famous of which is treatment with chromium solutions [23].

It is known [24] that biodegradable (absorbable) collagen has been used to treat wounds, close grafts and tooth extraction sites, as well as to accelerate recovery. Collagen-based membranes are also used as barriers in the treatment of periodontal diseases and implants to limit epithelial migration and allow cells with the ability to regenerate to fill the problem area. In some studies [25], collagen was injected in the form of hydrolysate (90%), while in others, in bovine form (2.3%) and pig form (3.4%). It has been found that collagen supplements provide better results in terms of improving the health of the skin, cornea, bones, gums, face, etc. Thus, the presented data confirm the importance and relevance of a detailed study of changes in the collagen of a bone implant during sterilizing treatment, therefore, in this work, the effect of ozone, ionizing radiation on bone collagen was evaluated using IR spectroscopy [26].

The registered spectra provide information about all components of bone tissue. Protein and mineral components create intense signals; the peaks of interest were marked accordingly. Information was obtained both on the mineral (carbonate replacing apatite in the lattice, and phosphate from apatite itself) and on collagen (amide I, II and III; the first is the result of stretching the peptide bond C=O, the second is the result of mixed stretching C-N and bending N-H in the plane, and the third is also the result of mixed stretching C-N and bending N-H in the plane with an additional contribution of stretching C-Ca).

Fluctuations characteristic of the organic phase (collagen) correspond to the fluctuations of amide groups (A and B, I, II, III) and the fluctuations of the C-H bond of aliphatic groups (CH, ${\mathrm{CH}}_{2}$ and ${\mathrm{CH}}_{3}$). The peaks characteristic of HAP correspond to fluctuations in the orthophosphoric groups of ${\mathrm{PO}}_{4}^{3-}$, hydroxide anions, carbonate ions ${\mathrm{CO}}_{3}^{2-}$ (in the case of carbonate-substituted HAP) and water. There are two bands near 621 cm^−1^ and 532 cm^−1^, which are associated with the content of apatite and acid phosphate, respectively. ${\mathrm{PO}}_{4}^{3-}\nu_{4}$ appears near 561, 580 and 604 cm^−1^ and resembles crystalline apatite. 

The carbonate content is determined by the deconvolution of the ${\mathrm{CO}}_{3}^{2-}\nu_{2}$ contour using three sub-ranges located near 879, 871 and 866 cm^−1^. The first two bands indicate apatite locations of the carbonate ion in two anionic sites of the structure, usually occupied by phosphate and hydroxyl ions, respectively. Thus, it is possible to distinguish B-type apatite (Figure 2). The main results of the IR spectroscopy, including the compounds that make up bone collagen, are presented in Table 1.

**Figure 5:**
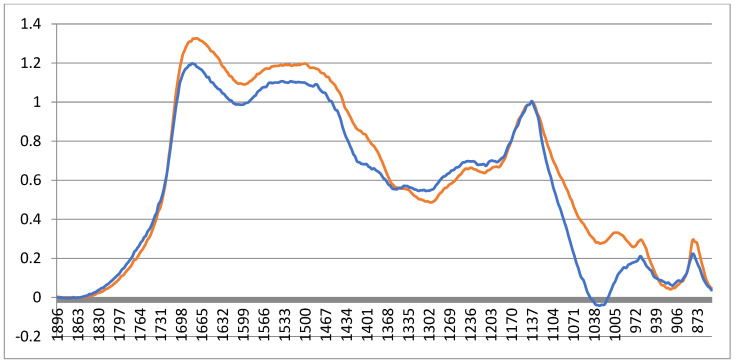
Results of studies on the IR spectrometer: spectra of the control sample (orange); samples after irradiation at 20 kGy (blue).

Important conclusions can also be drawn based on the analysis of changes in the spectra at different values of the dose load under radiation exposure (see Figure 5).

The amide 1 and amide 2 bands (bone collagen) are significantly reduced after exposure to an absorbed dose of 20 kGy, which confirms the need to reduce the standard dose.

**Figure 6:**
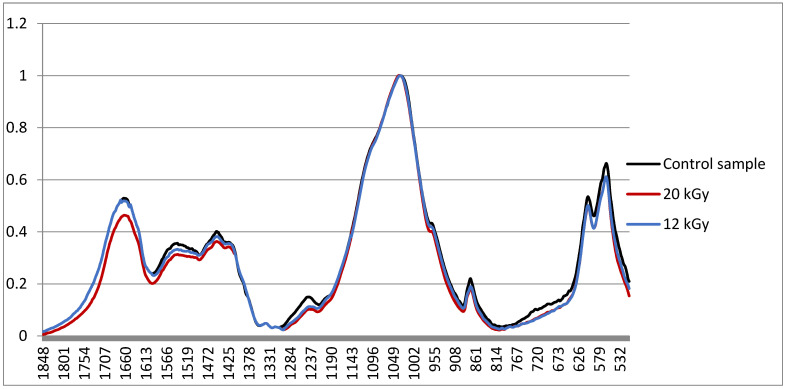
Results of studies on the IR spectrometer: spectra of the control sample (blue); samples after irradiation at 12 kGy (black) and at 20 kGy (red).

Figure 6 shows the data obtained for bone tissue samples subjected to successive ozone and radiation exposure during combined sterilization. The IR spectra for a control sample that has not been subjected to any treatment are shown in black, the spectra for bone tissue samples after successive ozone and radiation exposure with a dose of 12 kGy are in blue, and those with a dose of 20 kGy in red. The analysis of these results indicates that at a dose of 12 kGy, no significant changes were recorded compared to the control. After irradiation with a dose of 20 kGy, peaks decrease, and the content of bone collagen changes with an increase in the radiation dose, which affects the properties and quality of the bone.

The study of the patterns of changes in the structural and functional characteristics of the surface layer (microhardness) of bone fragments after various types of sterilizing effects in the above ranges of variation of sterilization parameters (ozone concentration in the ozone–oxygen mixture, the amount of absorbed dose during radiation treatment) and the generalization of the data obtained by the authors of [8,16,21,22,23] allow us to get an idea of the trends of changes occurring in the surface layers and the average results characterizing them (see Table 2).

The analysis of the presented data allows us to conclude that the selective sterilizing effect of an ozone–oxygen mixture with an ozone concentration of (6–8) mg/L lasting (15–20) min and radiation up to the absorbed dose of 20 kGy, as well as their combined sequential effect, do not lead to significant changes in the elastic-plastic characteristics of the bone fragment surface. At the same time, when processing a bone sample with ionizing radiation with an absorbed dose of 25 kGy (both separately and in combination with ozone exposure), there is a tendency for the microhardness index to decrease. These data correspond to the results of other researchers [27].

## 3. Materials and Methods

The experiments used samples obtained from fragments of the diaphysis of a femur from bovine, obtained via the author’s technology using hollow cutters [28]. The obtained samples were divided into 3 batches and subjected to various sterilizing effects: a selective ozone–oxygen mixture with a concentration of (6–8) mg/l for (15–20) minutes (the first batch), as well as irradiation with a stream of fast electrons (the second batch). The absorbed dose was varied in the range (5–25) kGy. Before carrying out these effects, the samples were placed in an airtight package. The samples of the third batch were subjected to a combined sterilizing effect by two of these factors, with the same parameters used in the following sequence: an ozone–oxygen mixture at the first stage and an ionizing effect at the second. Native samples without ozone–oxygen or radiation treatment were accepted as control samples.

The bone samples were examined using infrared spectroscopy (IR), scanning electron microscopy (SEM) with a prefix for elemental analysis, and atomic force microscopy, while mechanical analysis was used to determine elastic-plastic properties (Vickers microhardness index). Microbiological studies were conducted to assess the sterility of bone samples from the three batches.

The ozone–oxygen mixture was obtained using an industrial medical ozone generator Ac-GOXF-5-02-OZONE (JSC Electric Machine-building Plant “Lepse”, Kirov, Russia), which was supplied with oxygen from the VisionAire oxygen concentrator (Long Beach, CA, USA). The concentration of ozone in the ozone–oxygen working mixture was recorded in real time by the ozone concentration meter ICO-50 (JSC Electric Machine-building Plant “Lepse”, Kirov, Russia). Hermetic packing of bone samples was carried out using a two-layer thermal film and a sealing device F70-400 (Venlo, The Netherlands).

The samples were irradiated at the Lomonosov Moscow State University Institute of Nuclear Physics using a linear electron accelerator, which provides an electron beam with an energy of 1 MeV and an adjustable current in the range of 5 nA to 25 mA. Absorbed doses were 2, 5, 12, 15, 20 and 25 kGy; the dosimetric control of the radiation dose was carried out using dosimetric detectors—polymer films of size (10–12) × (30–35) mm, hermetically packed in 3 pieces, in compliance with the requirements of the instructions. During the experiment, the charge acting on the plate, the irradiation time, as well as the beam current were recorded. These indicators were used to estimate the amount of absorbed dose.

The analysis of structural and functional changes in the surface layer of bone samples was performed using a high-resolution scanning electron microscope (SEM) JSM-7800F 2012 (“Japanese Electron Optics Laboratory”—“JEOL”, Tokyo, Japan), which allows the simultaneous recording of the state of the structure of the surface layer and its elemental composition.

In the study of bone samples, vibrational spectra obtained using a Fourier transform infrared spectrometer Varian 7000 FTIR were used.

The studies of the mechanical characteristics of the surface layer of bone samples consisted in determining the Vickers microhardness index using a DM8 digital microhardness meter (Varese, Italy). The duration of the micro-action on the sample was 10 s with a load on the indenter of 50 g (0.490 N). The number of measurements of the microhardness index was determined taking into account the coefficient of variation and the maximum value of the confidence interval of 5%, used in many biomechanical works. The statistical processing was carried out using the standard program Statistica 13.3.

These studies were carried out at the experimental bases of Lomonosov Moscow State University, Tomsk Polytechnic University, All-Russian Research Institute of Medicinal and Aromatic Plants, North-Eastern Federal University, the Arctic Innovation Center, and the A. A. Borisyak Paleontological Institute, RAS.

## 4. Conclusions

A set of studies was carried out to establish the patterns of changes in the characteristics of the surface of bone implants as a result of combined sterilizing effects (ozone–oxygen mixture at the first stage and radiation at the second stage). This combined technology makes it possible to achieve the necessary sterilizing effect by reducing the dose load from ionizing radiation to values of 11–12 kGy, at which there are no negative changes in the morphomechanical characteristics of bone tissue. The synergistic effect of exposure to various sterilizing factors is achieved by effectively weakening the population of pathogens and reducing their radioresistance during the initial ozone exposure, which enhances the subsequent effect of radiation. In addition, an increase in oxygen content after ozone treatment also enhances the effectiveness of radiation exposure.

The results of instrumental studies show that exposure to ozone at the first stage of the combined sterilization process does not lead to morphological changes in the surface, mechanical properties (microhardness), or the characteristics of bone collagen. Only the oxygen content increases significantly, which in turn contributes to an increase in the effectiveness of radiation exposure at the second stage of combined sterilization, and, consequently, an additional reduction in the dose load. Combined (ozone + radiation) exposure at an absorbed dose of 12 kGy does not lead to significant changes in the content of collagen in bone tissue. Noticeable changes in the collagen content were recorded at high (20 kGy) absorbed dose values under radiation exposure.

Thus, the results of the conducted studies confirm the prospects of using a combined (ozone + radiation) technology for the sterilization of bone implants, which allows for the preservation of the native properties of the bone-plastic material to the maximum extent while ensuring the necessary level of its sterility.

This technology can be used not only in the manufacture of bone implants, but also in such an important area as the preservation of fossils of ancient animals (for example, in current programs for the study of the Yakut mammoth), in which the preservation of collagen remains is of particular importance.

## Figures and Tables

**Table 1:** Predominant organic compounds in the sample.

Spectra	Organic Compounds
1673 cm^−1^	absorption of the functional group of amide I (peptide C=O, stretching the vibration of collagen), which is an organic bone matrix (type I collagen)
1504.25 cm^−1^, 1450.89 cm^−1^, 1423.23 cm^−1^	the amide II band is caused by a combination of C–N stretching and N–H bending modes
1029.29 cm^−1^	corresponds to the normal modes v1 and v3 of the apatite phosphate ion
871.9 cm^−1^	carbonate v2
562.74 and 603.4 cm^−1^	corresponds to the bending mode v4 of the apatite phosphate ion.

**Table 2:** The value of the microhardness of the surface layer of the bone fragment after various types of sterilizing effects.

№	Type and Parameters of Bone Fragment Processing	Microhardness, MPa (M ± SD)
1	Native dry bone (control)	553 ± 10
2	Ozone–oxygen mixture (6–8 mg/L)	537 ± 14
3	Radiation—10 kGy	539 ± 15
4	Ozone–oxygen mixture + radiation—10 kGy	544± 19
5	Radiation—15 kGy	538 ± 20
6	Ozone–oxygen mixture + radiation—15 kGy	557 ± 19
7	Radiation—20 kGy	547 ± 19
8	Ozone–oxygen mixture + radiation—20 kGy	558 ± 20
9	Radiation—25 kGy	530 ± 19
10	Ozone–oxygen mixture + radiation—25 kGy	518 ± 23

## Data Availability

Data will be made available on request.
